# Fear of progression subtypes and their influencing factors in patients with coronary heart disease: A cross-sectional study using latent profile analysis

**DOI:** 10.1097/MD.0000000000048319

**Published:** 2026-04-24

**Authors:** Kai Bai, Weidong Huang, Jundan Luo, Zhiwen Deng, Shule Liu, Meiyi Yang, Jianan Tian, Yongduo Ma

**Affiliations:** a School of Nursing, Changchun University of Chinese Medicine, Changchun, Jilin, China.

**Keywords:** coronary heart disease, fear of progression, influencing factors analysis, potential profile analysis

## Abstract

This study analyzed the current status of fear of progression (FoP) in patients with coronary heart disease (CHD) and its latent profile categories, and explored the influencing factors of these different categories. A convenience sampling method was used to select CHD patients who were hospitalized in a tertiary hospital in Changchun City from October 2024 to June 2025 as the survey subjects. The general information questionnaire, the Chinese version of the simplified Fear of Disease Progression Scale, the Family Care Index Scale, the Pain Digital Evaluation Scale, and the Social Support Rating Scale were used for investigation. The potential profiles of FoP in CHD patients were analyzed, and the influencing factors were explored using univariate analysis and multivariate logistic regression. A total of 400 subjects were included in the survey. FoP in CHD patients could be divided into 3 latent profile categories: “Low-Risk Fear Type” (36.56%), “High-Risk Fear Type” (32.68%), and “Severe Fear Type” (30.76%). The influencing factors included pain, family per capita monthly income, family care, and chronic complications (all *P* < .05). The level of FoP in CHD patients is high and heterogeneous. Medical staff should focus on patients with a high-risk of fear and severe fear, and provide targeted prevention and psychological nursing for FoP in CHD patients as soon as possible, according to the characteristics and influencing factors of the different categories.

## 1. Introduction

Coronary heart disease (CHD) refers to heart disease caused by coronary atherosclerosis leading to narrowing, occlusion, or spasm of the coronary arteries, resulting in myocardial ischemia, hypoxia, or necrosis.^[1]^ The prevalence and mortality risk of CHD increase significantly with age.^[2]^ In the United States, the prevalence of CHD among individuals aged 20 and older is 6.4%.^[3]^ In European Union countries, approximately 40% of all deaths are attributed to CHD.^[4]^ According to the “Report on Cardiovascular Health and Diseases in China 2023,” the number of individuals with cardiovascular diseases in China has reached 330 million, with approximately 11.39 million suffering from CHD.^[5]^ Although advancements in diagnostic and treatment technologies and secondary prevention strategies have improved survival rates, the recurrence, rehospitalization, and multimorbidity associated with CHD remain common, correlating with higher long-term mortality risks and diminished quality of life.^[6]^

Fear of progression (FoP) refers to an individual’s fear concerning the potential progression or recurrence of a disease and its associated adverse physical, psychological, or social consequences.^[7]^ Research indicates that mild fear can elicit functional responses beneficial to health, such as promoting self-care or healthy behaviors.^[8]^ However, severe fear can lead to anxiety, depression, post-traumatic stress disorder, and other issues, adversely affecting subjective well-being, quality of life, and social functioning, and may even pose life-threatening risks requiring clinical intervention.^[9]^ Compared to other chronic diseases, the FoP burden in cardiovascular disease patients is more prominent.^[10]^ A Studies indicate that approximately half of patients with coronary heart disease express concerns about disease recurrence or progression, and their related support needs are often unmet.^[11]^

A study by Chinese researchers found that 59.5% of CHD outpatient patients experience FoP, with passive coping strategies exacerbating fear.^[12]^ Therefore, understanding the influencing factors of FoP in CHD patients and implementing corresponding interventions are crucial for their psychological health.^[13]^ However, existing studies have primarily classified FoP into functional and dysfunctional types based on total scale scores, neglecting individual heterogeneity.^[14]^ Latent profile analysis (LPA), a classification method based on latent variable theory, aims to identify subgroups with homogeneous characteristics by exploring response patterns across multiple latent continuous indicators.^[15]^ This approach facilitates the objective classification of different FoP subtypes in CHD patients and reveals differences among subtypes in demographic characteristics, disease-related indicators, and psychosocial factors.^[16]^ This study aims to classify FoP in CHD patients and analyze influencing factors, providing insights for targeted prevention and psychological care of FoP.

## 2. Materials and methods

### 2.1. Ethical statement and patient consent

This study was approved by the Medical Ethics Committee (approval number: CZDSFYLL2024XS-029), and all participants provided written informed consent.

### 2.2. Study design and participants

This study employed a convenience sampling method to select CHD patients hospitalized at a tertiary A-level hospital in Changchun City from October 2024 to June 2025 as survey subjects. The study adhered to the Strengthening the Reporting of Observational Studies in Epidemiology (STROBE) guidelines.^[17]^ Inclusion criteria: Patients who meet the diagnostic criteria for coronary heart disease and are in a stable condition after treatment; aged ≥ 18 years; clear consciousness and cognition, capable of normal communication. Exclusion criteria: Coexisting malignant tumors or severe organic diseases; mental disorders; significant speech or auditory-visual impairments. Exclusion criteria for data analysis: Incomplete data collection; withdrawal from the study during the investigation period. Based on LPA and sample size calculation methods for multivariate logistic regression, this study includes 20 items, comprising a demographic questionnaire (13 items) and 7 dimensions of relevant scales. Assuming a 20% missing rate, the minimum required sample size was calculated as N = (13 + 7) × 10/(1–20%) = 250.

### 2.3. Procedures

#### 2.3.1. General Information Questionnaire

This questionnaire was self-designed by the researchers and includes demographic data such as age, gender, personality traits, place of residence, educational level, marital status, number of children, average monthly household income, type of health insurance, and employment status. It also encompasses disease-related characteristics, including comorbid chronic conditions, family medical history, and history of percutaneous coronary intervention (PCI).

#### 2.3.2. Fear of Progression Questionnaire-Short Form (FoP-Q-SF)

Developed by Mehnert et al^[18]^ this scale is used to assess fear of disease progression in patients with cancer and chronic illnesses. Although the FoP-Q-SF was originally developed and validated in oncology populations, it has increasingly been applied in patients with chronic nonmalignant diseases, including cardiovascular conditions. It comprises 2 dimensions: physical health (6 items) and social/family functioning (6 items), totaling 12 items. Items 1 to 3, 5, 9, and 10 belong to the physical health dimension, while items 4, 6 to 8, 11, and 12 pertain to the social/family dimension. Each item is rated on a 5-point Likert scale. The total score ranges from 12 to 60, with higher scores indicating greater fear of disease progression. A score below 34 denotes functional fear of progression, whereas a score of 34 or above indicates dysfunctional fear of progression. The Chinese version of the scale has a Cronbach α coefficient of 0.883.

#### 2.3.3. Family APGAR Index (FAI)

This self-report questionnaire was developed by Smilkstein et al at the University of Washington in Seattle.^[19]^ It assesses an individual’s subjective perception of family caregiving and satisfaction with family functioning. The questionnaire consists of 5 items evaluating adaptation, partnership, growth, affection, and resolve. Each item is scored on a 3-point Likert scale: 0 (“almost never”), 1 (“sometimes”), and 2 (“almost always”). The total score ranges from 0 to 10, with higher scores indicating better family functioning. A total score of 7 to 10 suggests good family function, 4 to 6 indicates moderate dysfunction, and 0 to 3 denotes severe dysfunction. The overall Cronbach α coefficient for the scale is 0.90.

#### 2.3.4. Numeric Rating Scale (NRS) for pain

This scale requires patients to rate their pain intensity using an 11-point numerical scale from 0 to 10, where 0 indicates “no pain,” 1 to 3 represents “mild pain,” 4 to 6 denotes “moderate pain,” 7 to 9 signifies “severe pain,” and 10 corresponds to “worst possible pain.” The NRS is a straightforward and intuitive method for pain assessment, facilitating easy understanding, communication, and documentation. It significantly reduces the burden on healthcare providers and is widely used as a simple and effective evaluation tool.^[20]^

#### 2.3.5. Social Support Rating Scale (SSRS)

Developed by Xiao Shuiyuan in 1986, the SSRS is designed to assess an individual’s level of social support. The scale comprises 10 items across 3 dimensions: objective support (3 items), subjective support (4 items), and social support utilization (3 items). The total score ranges from 12 to 83, with higher scores indicating better perceived social support. The Cronbach α coefficient for the SSRS is 0.896.^[21]^

### 2.4. Data collection

Prior to the survey, the research team explained the study’s objectives and content to the participants and distributed the questionnaires. A standardized script was used to guide participants on how to complete the questionnaires and to address any questions they had. Disease and treatment-related information was collected by reviewing medical records and consulting with the patients. Upon completion, all questionnaires were immediately collected, and researchers checked for completeness, filled in any missing items, and clarified any illegible responses to ensure data quality. A total of 410 questionnaires were distributed, 10 of which were invalid, resulting in 400 valid responses, yielding a response rate of 97.56%.

### 2.5. Statistical analysis

Latent profile analysis was conducted using Mplus 8.4 (Muthen & Muthen, Los Angeles, www.statmodel.com) to identify latent profiles of FoP among CHD patients. The item scores from the FoP-Q-SF served as manifest indicators, and models with 1 to 4 profiles were sequentially tested. Model fit was assessed using the Akaike information criterion (AIC), Bayesian information criterion (BIC), adjusted BIC, entropy, the Lo–Mendell–Rubin adjusted likelihood ratio test (LMRT), and the bootstrap likelihood ratio test (BLRT). Lower AIC, BIC, and adjusted BIC values indicate better model fit; entropy values closer to 1 suggest more accurate classification; statistically significant LMRT and BLRT (*P* < .05) support the inclusion of additional profiles. The optimal model was selected based on these indices. Subsequently, statistical analyses were performed using SPSS 28.0 (IBM Corporation, Armonk; www.ibm.com). Descriptive statistics for continuous variables were presented as means and standard deviations or medians and interquartile ranges, while categorical variables were described using frequencies and percentages. Between-group comparisons based on the identified latent profiles were conducted using chi-square tests for categorical variables and the Kruskal–Wallis test for continuous variables. Multivariate logistic regression was employed to analyze factors influencing FoP, with *P* < .05 considered statistically significant.

## 3. Results

### 3.1. Current status and latent profile analysis results of FoP in patients with CHD

This study included 400 patients with CHD. The overall FoP score was 41.99 ± 6.791; the social/family dimension was 21.05 ± 3.496, and the physical health dimension was 20.94 ± 3.607. Starting from the initial model, 4 latent profile models were fitted, as shown in Table 1. As the number of latent classes increased, the AIC, BIC, and adjusted BIC values consistently decreased, indicating an improvement in model fit. LMR and BLRT results showed that the *P*-values for the 2-class and 3-class models were both <.001, suggesting that increasing the number of classes significantly enhanced model fit. However, the LMR test for the 4-class model was not significant (*P* = .556), indicating that the fit improvement was no longer significant when increasing from 3 to 4 classes. Furthermore, the entropy values for all models were high (>0.94), with the 4-class model having the highest entropy (0.979), indicating good classification clarity. Regarding class probability distribution, the 2-class model had proportions of 43.67% and 56.33%; the 3-class model had relatively balanced proportions (36.56%, 32.68%, 30.76%); and the 4-class model had distributions of 18.74%, 35.98%, 30.20%, and 15.08%. From the probability matrix of the 3 latent profile classes (Table 2), the average probabilities of class membership ranged from 96.9% to 99.2%. Considering the model fit indices, significance test results, and the interpretability of the classes, the 3-class model was selected as the final latent profile model (Table 1).

**Table 1:** Fit indices of the latent profile model for FoP in CHD patients.

Model	AIC	BIC	aBIC	LMRT	BLRT	Entropy	Potential profile proportion
1	10,708.360	10,804.155	10,728.001	–	–	–	–
2	8377.437	8525.121	8407.717	<0.001	<0.001	0.948	43.67%/56.33%
3	7729.979	7929.552	7770.899	<0.001	<0.001	0.959	36.56%/32.68%/30.76%
4	7434.682	7686.144	7486.241	0.556	<0.001	0.979	18.74%/35.98%/30.20%/15.08%

aBIC = adjusted Bayesian information criterion, AIC = Akaike information criterion, BIC = Bayesian information criterion, BLRT = bootstrap likelihood ratio test, CHD = coronary heart disease, FoP = fear of progression, LMRT = Lo–Mendell–Rubin adjusted likelihood ratio test.

**Table 2:** Average probability of each potential category.

Latent class	C1	C2	C3
C1	0.992	0.008	0.000
C2	0.011	0.969	0.020
C3	0.000	0.013	0.987

C1 = Low-Risk Fear Type, C2 = High-Risk Fear Type, C3 = Severe Fear Type.

### 3.2. Naming of the 3 latent profiles of FoP in patients with CHD

**Figure 1.**
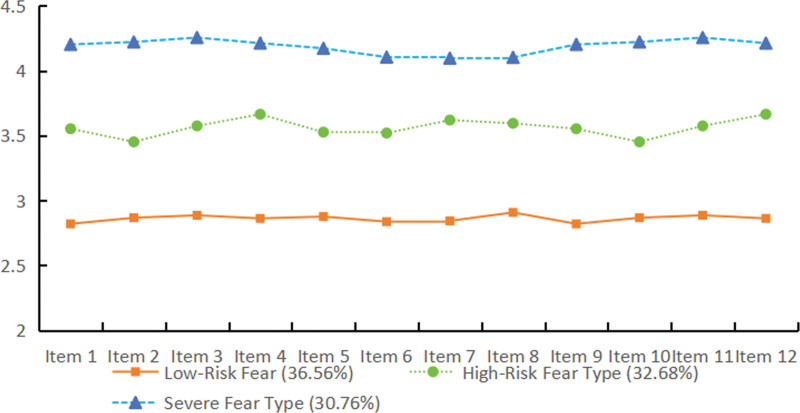
The 3 latent profile categories of FoP in CHD patients. CHD = coronary heart disease, FoP = fear of progression.

Based on the scores of the Chinese version of the Fear of Progression Questionnaire-Short Form (FoP-Q-SF) items across the 3 latent profile classes, the FoP latent profile diagram for CHD patients was constructed (see Fig. 1). Analyzing the score characteristics of each class: class 1 (C1) had the lowest average total score (~2.8), maintaining low levels across all indicators; class 2 (C2) had an average score of approximately 3.5, with overall moderate levels and minimal score variability among indicators, indicating a relatively stable trajectory; class 3 (C3) exhibited the highest scores among the 3 groups, with an overall mean around 4.2, and scores on all 12 indicators were at relatively high levels with minimal fluctuations. Therefore, the classes were sequentially named as follows: C1: “Low-Risk Fear Profile”; C2: “High-Risk Fear Type”; C3: “Severe Fear Type.” These profiles accounted for 36.56%, 32.68%, and 30.76% of the study participants, respectively (see Fig. 1).

### 3.3. Univariate analysis of latent profile categories of FoP in patients with CHD

Significant differences were observed among the 3 latent profile categories of CHD patients in terms of personality traits, average monthly household income, number of chronic comorbidities, history of percutaneous coronary intervention (PCI), pain status, total FoP score, social/family dimension, physical health dimension, and overall family caregiving score (*P* < .05), as detailed in Tables 3 and 4.

**Table 3:** Comparison of demographic characteristics among different latent profile classes in the study population (n = 400).

Variable		n	Percentage (%)	C1	C2	C3	(χ^2^/H)	*P*
Age (median, IQR)		400	100.00%	62.00 (55.00, 70.00)	61.00 (53.00, 65.00)	60.50 (52.00, 65.25)	2.454	.293
Gender	Male	241	60.30%	86 (35.7%)	82 (34.0%)	73 (30.3%)	0.312	.855
	Female	159	39.80%	60 (37.7%)	50 (31.4%)	49 (30.8%)		
Personality trait	Extroverted	199	49.75%	76 (38.2%)	74 (37.2%)	49 (24.6%)	6.897	.032
	Introverted	201	50.25%	70 (34.8%)	58 (28.9%)	73 (36.3%)		
Residence	Rural	160	40.00%	57 (35.6%)	48 (30.0%)	55 (34.4%)	2.096	.351
	Urban	240	60.00%	8 (37.1%)	84 (35.0%)	67 (27.9%)		
Marital status	Unmarried	5	1.25%	2 (40.0%)	2 (40.0%)	1 (20.0%)	5.534	.237
	Married	378	94.50%	135 (35.7%)	123 (32.5%)	120 (31.7%)		
	Divorced/widowed	17	4.25%	9 (52.9%)	7 (41.2%)	1 (5.9%)		
Children	Yes	376	94.00%	135 (35.9%)	125 (33.2%)	116 (30.9%)	0.976	.614
	No	24	6.00%	11 (45.8%)	7 (29.2%)	6 (25.0%)		
Education level	Primary school or below	186	46.50%	69 (37.1%)	54 (29.0%)	63 (33.9%)	8.790	.186
	Junior high school	128	32.00%	43 (33.6%)	43 (33.6%)	42 (32.8%)		
	High school or vocational school	41	10.25%	17 (41.5%)	14 (34.1%)	10 (24.4%)		
	College or above	45	11.25%	17 (37.8%)	21 (46.7%)	7 (15.6%)		
Monthly household income	<3000 CNY	201	50.25%	60 (29.9%)	78 (38.8%)	63 (31.3%)	9.942	.042
	3000–6000 CNY	162	40.50%	72 (44.4%)	44 (27.2%)	46 (28.4%)		
	>6000 CNY	37	9.25%	14 (37.8%)	10 (27.0%)	13 (35.1%)		
Medical insurance type	Out-of-pocket	127	31.75%	45 (35.4%)	38 (29.9%)	44 (34.6%)	1.892	.756
	Rural and urban residents	201	50.25%	73 (36.3%)	69 (34.3%)	59 (29.4%)		
	Urban employees	72	18.00%	28 (38.9%)	25 (34.7%)	19 (26.4%)		
Employment status	Employed	41	10.25%	16 (39.0%)	10 (24.4%)	15 (36.6%)	2.266	.687
	Unemployed	203	50.75%	70 (34.5%)	71 (35.0%)	62 (30.5%)		
	Retired	156	39.00%	60 (38.5%)	51 (32.7%)	45 (28.8%)		
Number of chronic comorbidities	0	119	29.75%	55 (46.2%)	37 (31.1%)	27 (22.7%)	16.941	.010
	1	127	31.75%	45 (35.4%)	45 (35.4%)	37 (29.1%)		
	2	88	22.00%	29 (33.0%)	33 (37.5%)	26 (29.5%)		
	≥3	66	16.50%	17 (25.8%)	17 (25.8%)	32 (48.5%)		
Family history of CHD	Yes	231	57.75%	82 (35.5%)	74 (32.0%)	75 (32.5%)	0.999	.607
	No	169	42.25%	64 (37.9%)	58 (34.3%)	47 (27.8%)		
History of PCI	Yes	212	53.00%	66 (31.1%)	73 (34.4%)	73 (34.4%)	6.131	.047
	No	188	47.00%	80 (42.6%)	59 (31.4%)	49 (26.1%)		

C1 = Low-Risk Fear Type, C2 = High-Risk Fear Type, C3 = Severe Fear Type.

CHD = coronary heart disease, IQR = interquartile range, PCI = percutaneous coronary intervention.

**Table 4:** Comparison of NRS, FoP-Q-SF, FAI, and SSRS among different latent profile categories of FoP (mean ± SD).

Variables	M ± SD	Latent class			Post hoc comparison	H	*P*
		C1 (n = 146)	C2 (n = 132)	C3 (n = 122)			
Pain situation	3.00 (2.00, 4.00)	3.00 (2.00, 4.00)	3.00 (2.00, 4.00)	4.00 (3.00, 5.00)	C1 < C2 < C3	30.074	<.001
Total FoP score	42.00 (35.00, 48.75)	34.00 (33.00, 35.00)	43.00 (42.00, 44.00)	50.00 (49.00, 52.00)	C1 < C2 < C3	354.832	<.001
Social dimension	21.00 (18.00, 24.00)	17.00 (17.00, 18.00)	22.00 (21.00, 23.00)	25.00 (24.00, 26.00)	C1 < C2 < C3	333.648	<.001
Physical health dimension	21.00 (18.00, 24.00)	18.00 (16.00, 18.00)	21.00 (20.00, 22.00)	25.00 (24.00, 26.00)	C1 < C2 < C3	343.720	<.001
FAI total score	7.00 (4.00, 9.00)	9.00 (5.00, 10.00)	6.00 (5.00, 9.00)	6.50 (2.00, 8.00)	C1 > C2 = C3	25.044	<.001
SSRS total score	37.00 (34.00, 40.00)	37.00 (34.00, 40.25)	37.50 (34.00, 40.75)	38.00 (34.00, 40.00)	C1 = C2 = C3	0.158	.924
Utilization of support	9.00 (7.00, 9.00)	9.00 (7.00, 10.00)	8.50 (7.00, 10.00)	8.00 (7.00, 9.00)	C1 = C2 = C3	2.046	.359
Subjective support	19.00 (16.00, 22.00)	19.00 (16.00, 22.00)	18.00 (15.25, 22.00)	19.00 (17.00, 22.00)	C1 = C2 = C3	0.809	.667
Objective support	10.00 (9.00, 12.00)	10.00 (8.00, 11.25)	10.00 (9.00, 11.75)	10.00 (8.00, 12.00)	C1 = C2 = C3	1.505	.471

C1 = Low-Risk Fear Type, C2 = High-Risk Fear Type, C3 = Severe Fear Type, C1 = C2 = C3: no significant difference in the means across the different categories.

FAI = Family APGAR Index, FoP = fear of progression, FoP-Q-SF = Fear of Progression Questionnaire-Short Form, NRS = Numeric Rating Scale, SD = standard deviation, SSRS = Social Support Rating Scale.

### 3.4. Multivariate logistic regression analysis of latent profile categories of FoP in patients with CHD

A multinomial logistic regression model was employed with the latent profile categories of FoP in CHD patients as the dependent variable, assigning values of 1, 2, and 3 to the “Low-Risk Fear Profile,” “High-Risk Fear Type,” and “Severe Fear Type,” respectively. Variables with statistical significance in the univariate analysis were included as independent variables in the multivariate logistic regression model. The coding for these variables was as follows: personality traits (introverted = 0, extroverted = 1); monthly household income (<3000 CNY = 1, 3000–6000 CNY = 2, >6000 CNY = 3); number of chronic comorbidities (0 = 1, 1 = 2, 2 = 3, ≥3 = 4); history of percutaneous coronary intervention (PCI; no = 0, yes = 1). The variable selection criteria were set to the system default (inclusion α = 0.05, exclusion α = 0.10). The results indicated that pain was a common influencing factor across all 3 FoP categories (all *P* < .05). Higher monthly household income and better family functioning were specific protective factors for the High-Risk Fear Type (*P* < .05), while a greater number of chronic comorbidities was a specific risk factor for the Severe Fear Type (*P* < .05), as detailed in Table 5.

**Table 5:** Multivariable logistic regression results for FoP latent profile categories in CHD patients.

Group comparison	Variables	β	SE	Wald	*P*	OR	95% CI
C2 vs C1	Constant	−0.327	1.171	0.078	.780	0.721	
	Monthly household income per capita	−0.541	0.202	7.162	.007	0.582	0.392–0.865
	Family care dimension	−0.101	0.046	4.834	.028	0.904	0.825–0.989
	Pain situation	0.252	0.117	4.615	.032	1.287	1.022–1.620
C3 vs C1	Constant	−1.797	1.302	1.905	.168	0.166	
	Number of chronic comorbidities	0.280	0.133	4.449	.035	1.323	1.020–1.716
	Pain situation	0.510	0.135	14.293	.000	1.666	1.279–2.170
C3 vs C2	Constant	−1.459	1.177	1.538	.215	0.232	
	Pain situation	0.314	0.114	7.611	.006	1.369	1.095–1.710

C1 = Low-Risk Fear Type, C2 = High-Risk Fear Type, C3 = Severe Fear Type.

CHD = coronary heart disease, CI = confidence interval, FoP = fear of progression, OR = odds ratio, SE = standard error.

## 4. Discussion

### 4.1. Results of the FoP study in patients with CHD

The findings of this study indicate that patients with CHD exhibit elevated levels of FoP, with greater fear regarding their own disease and health than concerning social and familial functioning, consistent with previous research.^[22,23]^ This study identified 3 FoP subtypes in CHD patients: Low-Risk Fear Type, High-Risk Fear Type, and Severe Fear Type, which accounted for 36.56%, 32.68%, and 30.76% of the participants, respectively. The majority of CHD patients experience varying degrees of fear related to disease progression, with nearly one-third classified under the Severe Fear Type suggesting a significant prevalence of FoP among this population. This heightened fear may be attributed to CHD being a high-risk cardiovascular condition, where patients often experience immediate discomfort due to pain and increased challenges in disease management due to comorbidities, leading to a heightened perception of health deterioration risks.^[24,25]^

### 4.2. Characterization of patients with different FoP subtypes

In this study, patients in the Low-Risk Fear Type were mainly characterized by mild pain during disease episodes^[26]^; higher per capita monthly family income and better family functioning were specific protective factors for the High-Risk Fear Type^[27,28]^; the distribution of patients with 2 or more chronic comorbidities and severe pain during disease episodes in the Severe Fear Type was relatively high, which was similar to the results of domestic and international studies. Similar,^[29]^ it may be that patients in the Low-Risk Fear Type have better physical conditions, no history of chronic diseases, a lower disease burden, and mild onset symptoms, leading to better adaptation and a low-risk of FoP.^[30]^ For High-Risk Fear Type patients, a higher per capita monthly family income can reduce the economic burden of treatment, thereby preventing the fear of economic concerns from compounding the uncertainty of the disease and exacerbating fear. Additionally, good family functioning, through emotional support and assistance in disease management, helps build their confidence.

### 4.3. Analysis of factors influencing FoP in CHD patients

#### 4.3.1. Relationship between pain conditions and FoP in CHD patients

This study indicates that pain is a common influencing factor across all subtypes of FoP in patients with CHD. The underlying mechanism is that pain serves as the most direct physiological signal for CHD patients perceiving disease progression.^[31]^ Mild pain may trigger anxiety regarding disease recurrence, while moderate to severe pain is directly associated with serious consequences such as myocardial ischemia and infarction. Pain is not merely a physiological discomfort; it also exacerbates patients’ fear of disease deterioration and future health status.^[32]^ Therefore, healthcare providers should incorporate pain assessment as an integral part of routine monitoring for CHD patients, especially during the post-discharge or rehabilitation phases. Early identification and continuous monitoring of pain may facilitate the recognition of patients at higher risk of elevated FoP, thereby supporting timely intervention.

#### 4.3.2. Relationship between family income, family function, and FoP in CHD patients

The study indicates that, compared to the Low-Risk Fear Type, higher monthly household income and better family functioning are specific protective factors for the High-Risk Fear Type. This finding aligns with results from international studies.^[33]^ Higher family income may provide patients with more stable financial support, alleviating the economic burden associated with disease treatment and management.^[34]^ This financial stability enables patients to access better medical resources, pharmaceutical treatments, and rehabilitation support, thereby reducing the fear of disease progression. Furthermore, good family functioning involves support and interaction among family members, offering necessary emotional support and psychological comfort to patients. This support helps mitigate feelings of helplessness and anxiety in the face of illness.^[35,36]^ Research has shown that emotional support from the family can effectively alleviate patients’ fear of disease progression. Particularly in coping with long-term chronic diseases, the companionship and understanding of family members can significantly enhance patients’ psychological resilience and coping strategies.^[37]^ Therefore, it is recommended that healthcare providers, when addressing High-Risk Fear CHD patients, prioritize assessing their family economic status, explain healthcare policies, and provide information on chronic disease treatment subsidies and other financial support resources to alleviate disease-related economic pressures. Additionally, assessing family functioning levels and offering guidance on optimizing family functions, imparting emotional support techniques, and collaborative disease management methods to patients and their families are advisable.

#### 4.3.3. Relationship between chronic comorbidities and FoP in CHD patients

This study indicates that, compared to the Low-Risk Fear Type, patients with 2 or more chronic comorbidities are more likely to be classified into the Severe Fear Type. The presence of multiple chronic conditions leads to interrelated pathological states, increasing the incidence of adverse events in CHD patients.^[38,39]^ This exacerbates both the economic and psychological burdens on patients, thereby elevating the risk of FoP. Healthcare providers should prioritize attention to CHD patients with multiple comorbidities, enhance follow-up care and psychological support, and implement multidisciplinary nursing interventions to provide personalized comprehensive management, aiming to improve patients’ physical and mental health.

### 4.4. Clinical implications for screening and intervention

These findings have important clinical implications. For patients classified as the “Low-Risk Fear Type,” routine psychoeducation and periodic monitoring may be sufficient. In contrast, for those categorized as the “High-Risk Fear Type,” greater emphasis should be placed on assessing family economic status and family functioning. Targeted interventions may include facilitating access to financial assistance programs, involving social workers in care planning, and providing family-centered counseling to strengthen both emotional and practical support. For patients classified as the “Severe Fear Type,” who present with multiple comorbidities and a substantial pain burden, a multidisciplinary management approach may be particularly beneficial. Such an approach may involve coordinated collaboration among cardiologists, pain management specialists, and mental health professionals to ensure comprehensive and integrated care.

### 4.5. Limitations

The primary limitation of this study lies in its cross-sectional design and the use of a convenience sample recruited from a single tertiary hospital in Changchun City. These factors limit the generalizability of the findings to the broader population of patients with CHD in China and other regions. The single-center design may introduce selection bias, and therefore the results should be interpreted with caution across different healthcare settings, geographic regions, and socioeconomic backgrounds. Future research should employ multicenter designs with larger and more diverse samples to further validate and extend the present findings.

## 5. Conclusion

Through a cross-sectional survey, this study identified 3 latent subtypes of FoP in CHD patients. Pain experience, family income, family functioning, and chronic comorbidities were significantly associated with latent profile membership of FoP. Nurses should focus on patients classified as High-Risk Fear Type and Severe Fear Type and, based on the characteristics and influencing factors of each subtype, implement targeted prevention and psychological care to address the fear of disease progression at an early stage.

## Acknowledgments

The authors thank all the patients and staff who participated in this study.

## Author contributions

**Conceptualization:** Kai Bai.

**Data curation:** Jundan Luo.

**Funding acquisition:** Yongduo Ma.

**Investigation:** Zhiwen Deng, Meiyi Yang.

**Methodology:** Jianan Tian.

**Supervision:** Weidong Huang.

**Validation:** Weidong Huang.

**Writing – original draft:** Shule Liu.

**Writing – review & editing:** Kai Bai.
